# The BDNF effects on dendritic spines of mature hippocampal neurons depend on neuronal activity

**DOI:** 10.3389/fnsyn.2014.00005

**Published:** 2014-03-20

**Authors:** Yves Kellner, Nina Gödecke, Tobias Dierkes, Nils Thieme, Marta Zagrebelsky, Martin Korte

**Affiliations:** Division of Cellular Neurobiology, Zoological Institute, TU BraunschweigBraunschweig, Germany

**Keywords:** dendrites, spines, structural plasticity, hippocampus, neurotrophins

## Abstract

The fine tuning of neural networks during development and learning relies upon both functional and structural plastic processes. Changes in the number as well as in the size and shape of dendritic spines are associated to long-term activity-dependent synaptic plasticity. However, the molecular mechanisms translating functional into structural changes are still largely unknown. In this context, neurotrophins, like Brain-Derived Neurotrophic Factor (BDNF), are among promising candidates. Specifically BDNF-TrkB receptor signaling is crucial for activity-dependent strengthening of synapses in different brain regions. BDNF application has been shown to positively modulate dendritic and spine architecture in cortical and hippocampal neurons as well as structural plasticity *in vitro*. However, a global BDNF deprivation throughout the central nervous system (CNS) resulted in very mild structural alterations of dendritic spines, questioning the relevance of the endogenous BDNF signaling in modulating the development and the mature structure of neurons *in vivo*. Here we show that a loss-of-function approach, blocking BDNF results in a significant reduction in dendritic spine density, associated with an increase in spine length and a decrease in head width. These changes are associated with a decrease in F-actin levels within spine heads. On the other hand, a gain-of-function approach, applying exogenous BDNF, could not reproduce the increase in spine density or the changes in spine morphology previously described. Taken together, we show here that the effects exerted by BDNF on the dendritic architecture of hippocampal neurons are dependent on the neuron's maturation stage. Indeed, in mature hippocampal neurons *in vitro* as shown *in vivo* BDNF is specifically required for the activity-dependent maintenance of the mature spine phenotype.

## Introduction

Neurotrophins are essential for multiple aspects of neuronal development and function. Especially Brain-derived Neurotrophic Factor (BDNF) has been shown to play an important role in neuronal survival and in the maintenance of several neuronal systems. In addition, BDNF has been implicated in numerous processes of functional and structural synaptic plasticity (Gottmann et al., 2009; Park and Poo, 2012; Zagrebelsky and Korte, 2013). The role of BDNF has been extensively analyzed *in vitro* upon the application of exogenous BDNF. The data obtained support the notion that during development BDNF is involved in regulating the fine-tuning of the cortical network by selectively enhancing dendritic growth in an activity-dependent manner (Mcallister et al., 1995, 1996; Horch et al., 1999). Application of exogenous BDNF to developing primary hippocampal neurons has been shown to result in a significant increase in the number of primary neurites as well as an increase in neurite complexity and length (Ji et al., 2005; Kwon and Sabatini, 2011). Furthermore, dendritic spine density and morphology of mature primary hippocampal neurons are significantly influences by a BDNF application (Ji et al., 2005, 2010). Similarly, mature organotypic hippocampal neurons treated with BDNF show a significant increase in dendritic spine density and in the number of synapses (Tyler and Pozzo-Miller, 2001, 2003). Taken together, the *in vitro* studies described above strongly support the notion that, in the hippocampus exogenous BDNF promotes dendritic formation and growth during development and regulates dendritic spine density and morphology in mature neurons. But as strong as the evidence for a role of BDNF in modulating dendritic architecture might appear, the preparation techniques (Danzer et al., 2004) and the culture conditions (Chapleau et al., 2008) have been shown to influence the expression levels as well as the cellular response to BDNF, possibly confounding the analysis under these conditions. Suggestive for a role of BDNF *in vivo* is the correlation between the physiological variability in BDNF expression levels in the mouse dentate gyrus and the dendritic spine density in granule cells (Stranahan, 2011). Moreover, a reduction in BDNF serum levels is associated to a reduction in hippocampus volume in aging humans (Erickson et al., 2011) as well as in dendritic complexity and spine density in senescent rats (Von Bohlen Und Halbach, 2010). The *in vivo* role of BDNF has been very difficult to evaluate in the post-natal brain as *bdnf^−/−^* mouse mutants die too early for the role of BDNF to be assessed after its increased post-natal expression caused by neuronal activity (Zafra et al., 1990; Hong et al., 2008). The analysis of *bdnf^+/−^* mouse mutants, showing reduced BDNF levels provided evidence that, *in vivo* BDNF plays an important role in the induction of LTP in the hippocampus (Korte et al., 1995), the acquisition of extinction learning (Psotta et al., 2013) and in the structural rearrangement of adult cortical circuitry upon increased sensory input (Genoud et al., 2004). On the other hand, in *bdnf^+/−^* mice synapse density and spine morphology are indistinguishable from those in WT mice (Korte et al., 1995; Genoud et al., 2004) and a compensatory increase in TrkB receptor expression occurs (Carreton et al., 2012), leaving open the question of whether BDNF modulates dendritic architecture *in vivo*. Thus, a number of studies have been performed using different conditional gene targeted mouse lines and Cre-loxP-mediated excision of *bdnf* (Rios et al., 2001; Gorski et al., 2003; Baquet et al., 2004; He et al., 2004; Chan et al., 2006, 2008; Monteggia et al., 2007; Unger et al., 2007; Rauskolb et al., 2010). Surprisingly, the effect on excitatory neurons in the hippocampus and cortex of a global BDNF deprivation throughout the central nervous system (CNS) is extremely mild when compared to the effects observed upon a BDNF application *in vitro*. Rauskolb et al. (2010) showed that in a Tau-BDNF^KO^ mouse the volume of the cortex is only slightly reduced and the one of the hippocampus unchanged. Accordingly, dendritic complexity of CA1 pyramidal cells is only mildly reduced and while no changes could be observed in dendritic spine density the spine type distribution in Tau-BDNF^KO^ neurons is significantly shifted toward a more immature phenotype, pointing to a specific role of BDNF in the maintenance of mature dendritic spines in the mature hippocampus. These results are consistent with the observation that a conditional deletion of TrkB in the hippocampus does not affect the gross dendritic morphology of CA1 pyramidal neurons (Minichiello et al., 1999; Luikart et al., 2005).

So overall it is noteworthy that while most of the published data supporting a role for BDNF in modulating dendrite and dendritic spine morphology derive from *in vitro* experiments applying exogenous BDNF, a role for endogenous BDNF in this context is still unclear. Moreover, *in vivo* the effects of endogenous BDNF in modulating the structure of neurons seem to be extremely specific, depending on the developmental stage, the brain area as well as the cell-type. Therefore, in this study we set out to address the discrepancies in the role of BDNF in modulating the architecture of mature hippocampal neurons. In order to contribute to a better understanding of the BDNF activity, we analyzed the effects of several manipulations of its signaling on dendrites and dendritic spines at different developmental stages and under different levels of neuronal activity in primary neuronal cultures. The results we obtained in our culture system confirm the observations previously obtained *in vivo* and show that BDNF exerts a highly precise role on different aspects of neuronal architecture in an age-dependent manner. In mature hippocampal neurons *in vitro*, as shown *in vivo* endogenous BDNF is specifically required for the activity-dependent maintenance of the mature spine phenotype.

## Materials and methods

### Preparation of primary hippocampal cultures

Primary hippocampal cultures were prepared from C57BL/6 mice at embryonic day 18. All procedures were approved by guidelines from the Animal Committee on Ethics in the Care and Use of Laboratory Animals of TU Braunschweig. Embryos were decapitated and the brains were kept in ice-cold Gey's balanced salt solution (GBSS) containing of 1% kynurenic acid, 1% glucose and adjusted to pH 7.2. The dissected hippocampi were incubated for 30 min in trypsin/EDTA at 37°C and then mechanically dissociated. Cells were plated at high density (10^5^/well) on poly-L-lysine-coated coverslips (13 mm) and kept in Neurobasal medium (Invitrogen) supplemented with 2% B27 (Invitrogen) and 0.5 mM Glutamax at 37°C, 5% CO_2_, and 99% humidity. For analysis of neurite growth in 3–6 days *in vitro* to the cells were plated at a density of 10^4^ cells per well. For the experiments performed in high Magnesium (Mg^2+^) containing medium, the cells were plated at a density of 70,000 cells per well and kept in Neurobasal medium (Gibco) supplemented with 2% BSA (Gibco), 200 μ M L-Glutamin and 1% 100 × N2 (Invitrogen) at 37°C, 5% CO_2_, and 99% humidity.

### Transfection of primary hippocampal neurons

Hippocampal neurons were transfected at various time points with a farnesylated form of the enhanced green fluorescent protein (fEGFP) using Lipofectamine 2000 according to the manufacturer's protocol. To specifically label F-actin without adverse effects on actin dynamics the cells were co-transfected with fEGFP and an expression plasmid for the Lifeact peptide fused to a red fluorescent protein (RFP-LA) (Riedl et al., 2008, 2010). Briefly, 0.8 μ g DNA or, for a co-transfection 0.4 μg pfEGFP and 0.6 μg pRFP-LA and 2 μ l Lipofectamine 2000 per well were mixed in 100 μl Neurobasal medium, and added to the cultures drop-wise. After 50 min of incubation the transfection medium was exchanged for the original Neurobasal medium. The cultures were kept for 24 h at 37°C, 5% CO_2_, and 99% humidity before the treatment.

### Treatment of primary hippocampal neurons

Twenty-four hours after transfection, 1/3 of the culture medium was exchanged for a medium containing 0.1% Bovine Serum Albumine (BSA, Sigma; 0.1% BSA/PBS) as a control treatment or 0.1% BSA combined with BDNF (Recombinant Human BDNF, 40 ng/ml, R&D Systems), BDNF blocking antibodies (BDNF-Abs, 1:500, M. Sendtner, Wuerzburg Germany) or TrkB receptor bodies (TrkB-Fc, Recombinant Human TrkB-Fc, 500 ng/ml, R&D Systems). The BSA was added to the medium to prevent BDNF or the antibodies from sticking to the plastic (Chen et al., 1999). In a second set of control experiments the cultures were kept in Neurobasal medium without addition of BSA. As no significant difference between the two control conditions could be observed only the BSA control was used in the result part. The cultures were fixed 24 h after the treatment in 4% paraformaldehyde (PFA) in 0.1 M phosphate buffer (PBS), washed and mounted with an anti-fading aqueous mounting medium (Fluoro-Gel Emsdiasum).

In a second set of experiments neuronal activity was reduced in primary hippocampal cultures by increasing the Mg^2+^ concentration in the medium. Indeed, Mg^2+^ decreases the activation of voltage-gated channels thereby reducing neuronal excitability (Mayer and Westbrook, 1987; Dribben et al., 2010). To avoid effects on the early developmental stages Mg^2+^ concentration in the medium was increased starting at 7 DIV from 1.5 to 3.5 mM by adding MgCl_2_, for 2 weeks and fresh medium was added once a week. The treatment with BDNF or TrkB receptor bodies was performed for 24 h either in low (1.5 mM) or high (3.5 mM) Mg^2+^ containing medium.

### Immunocytochemistry

Primary hippocampal cultures (6 or 23 DIV) were fixed with 4% PFA, permeabilized and blocked with a solution containing 0.2% Triton X-100, 10% goat serum, and 0.1% BSA in PBS. A rabbit polyclonal antibody anti-c-fos (Santa Cruz Biotechnology, Inc., sc-52; dilution 1:500), a mouse monoclonal antibody anti-CaMKII (Invitrogen, dilution 1:500), a rabbit polyclonal anti-MAP2 (Abcam, dilution 1:500) or a primary rabbit monoclonal anti-phospho-TrkA (Tyr674/675)/TrkB (Tyr706/707) antibody (Cell Signaling Technology; dilution 1:200) were diluted in blocking solution (10% goat serum and 0.1% BSA in PBS) and incubated at 4°C overnight. Secondary anti-rabbit or anti-mouse antibodies conjugated with Cy2, Cy3, or Cy5 (Jackson Immuno Research) were diluted 1:500 in PBS and incubated for 2 h at room temperature. Alexa Fluor 350 phalloidin (Lifetechnologies) was diluted 1:50 in PBS and incubated for 3 h at room temperature to selectively stain F-actin. Finally, cultures were counterstained with DAPI (4′,6-diamidino-2-phenylindole) (Biotium) diluted 1:1000 in PBS for 5 min, washed and mounted using an anti-fading aquous mounting medium (Fluoro-Gel Emsdiasum).

### Neuron selection

The transfection of the fEGFP expression plasmid under the control of the CMV promoter resulted in the intense labeling of the entire dendritic arbor as well as of all dendritic spines of individual pyramidal neurons. A small number of individual pyramidal neurons were transfected in each culture allowing to following the complete dendritic processes of each labeled neuron. Only non-overlapping labeled neurons were selected for our analysis allowing for an unambiguous reconstruction of the entire dendritic tree as well as the analysis for the spine density. Degenerating neurons were identified according to the presence of retraction bulbs and fragmentation of the dendritic processes and excluded from the analysis. Due to a recent report showing that Mg^2+^ (5 to 10 mM) increases neuronal apoptosis in primary hippocampal neurons (Dribben et al., 2010), special care was used in selecting neurons kept in high magnesium medium (3.5 mM). Indeed, all neurons selected for the analysis showed a healthy cell body and dendritic tree with no signs of apoptosis.

### Neuronal imaging and analysis

The neurons selected for analysis were imaged using an Axioplan 2 microscope equipped with an ApoTome module (Zeiss). Each neuron was first imaged using a 20× objective [1.25 numerical aperture (NA)] and the images were used to analyze dendritic complexity and length using a Sholl analysis (Sholl, 1953). The neurons were traced using the Neurolucida software (MicroBrightField), and the Sholl analysis was obtained with Neuroexplorer software (MicroBrightField), calculating the cumulative number of dendritic intersections at 10 μm interval distance points starting from the cell body. The total number of crossings for each cell was used as an index for total dendritic complexity. The total neurite length, number of primary neurites and number of branching points of 6 DIV neurons were analyzed by tracing MAP2 labeled neurites using the NeuronJ (Meijering et al., 2004) plugin for ImageJ (NIH) Software. Spine density was measured separately for portions of selected second or third order dendrite branches and imaged using a 63× objective (1.32 NA) acquiring three-dimensional stacks z-sectioned at 0.5 μm. The number of spines and the dendritic length were measured using the ImageJ software. The number of spines was normalized per micrometer of dendritic length and a minimal length of 250 μm of dendrite per cell was analyzed. The maximal spine head diameter and maximal spine length (from the dendritic shaft to the spine head tip) were measured on three-dimensional stacks using the ImageJ (NIH) software. To examine the actin content in dendritic spine heads the mean fluorescent intensity of RFP-lifeact was measured with the area selection tool of ImageJ and normalized to the mean fluorescent intensity of the dendritic shaft (for normalization of the RFP overexpression, Figure 3B, indicated with the arrow heads). RFP-Lifeact and the immunocytochemistry for c-fos and phosphorylated TrkB were imaged keeping a constant exposure time. The mean fluorescent intensity values for the immunocytochemistry of c-fos and phosphorylated TrkB were measured with ImageJ (NIH) and normalized to the control condition. The analysis was performed blindly.

### Calcium imaging

Primary hippocampal cultures 16 and 23 DIV were loaded with calcium indicator by incubating them for 15 min with the indicator solution (50 μg Oregon Green 488 BAPTA-1 AM (Invitrogen), dissolved in 50 μl DMSO and 2.5% Pluronic acid) at 37°C, 5% CO_2_, and 99% humidity. The cells were then transferred to HBSS (Hanks balanced salt solution) and kept 45 min at 37°C, 5% CO_2_, and 99% humidity. The coverslips were thentransferred to the recording chamber, continuously perfused with HBSS and kept at 32°C. The live imaging was performed using an Olympus BX61WI microscope system (Cell^∧^M software) and a CCD camera (VisiCam QE, Visitron Systems). Images were recorded at 6 Hz for 75 sec with an Olympus 20× water immersion objective (0.95 NA XLUMPLAN FL, Olympus), whereby a pixel resolution of 0.77 μm/pixel was achieved. A frame rate of 150 ms and an exposure time of 61 ms were used. Primary hippocampal cultures were treated with BSA (serial 0.1% BSA/1× PBS) or BDNF (Recombinant Human BDNF, 40 ng/ml, R&D Systems) by a 5 min bath-application.

The analysis of calcium transients was performed using ImageJ (NIH) and calculating the fluorescence changes as average of all pixels within regions of interests (ROIs). The ROIs were set around the cell bodies of the neurons. For the background subtraction the ROI was drawn on a neighboring area containing neuropil. The resulting changes in the intracellular calcium concentration are represented as Δ*F*/*F*_0_ in percent, where *F*_0_ is the fluorescence at resting conditions [Δ*F*/*F*_0_ = (*F* − *F*_0_)/*F*_0_] taken before the application of BDNF (Lang et al., 2006). In addition to it we generated using the plugin “reslice” of the ImageJ Software a pseudo-line scan to visualize changes in the fluorescence intensity for the whole Δ*F*-stack. In the pseudo-line scan the y-axis corresponds to the respective line drawn on the stack whereas the x-axis corresponds to time.

### Statistical analysis

The statistical analysis was performed using Microsoft Excel or GraphPad Prism. The data obtained were compared between two different experimental conditions using an unpaired two-tailed Student's *t*-test. Data including more than 2 different groups were analyzed using a One-Way ANOVA followed by a *post-hoc* Tukey's Multiple Comparison Test. Values of *p* = 0.05 were considered significant and plotted as follows ^*^p < 0.05; ^**^p < 0.01; ^***^p < 0.001. All data are indicated as mean ± s.e.m.

## Results

### Endogenous BDNF regulates dendritic spine density and morphology in mature primary hippocampal neurons

**Figure 1:**
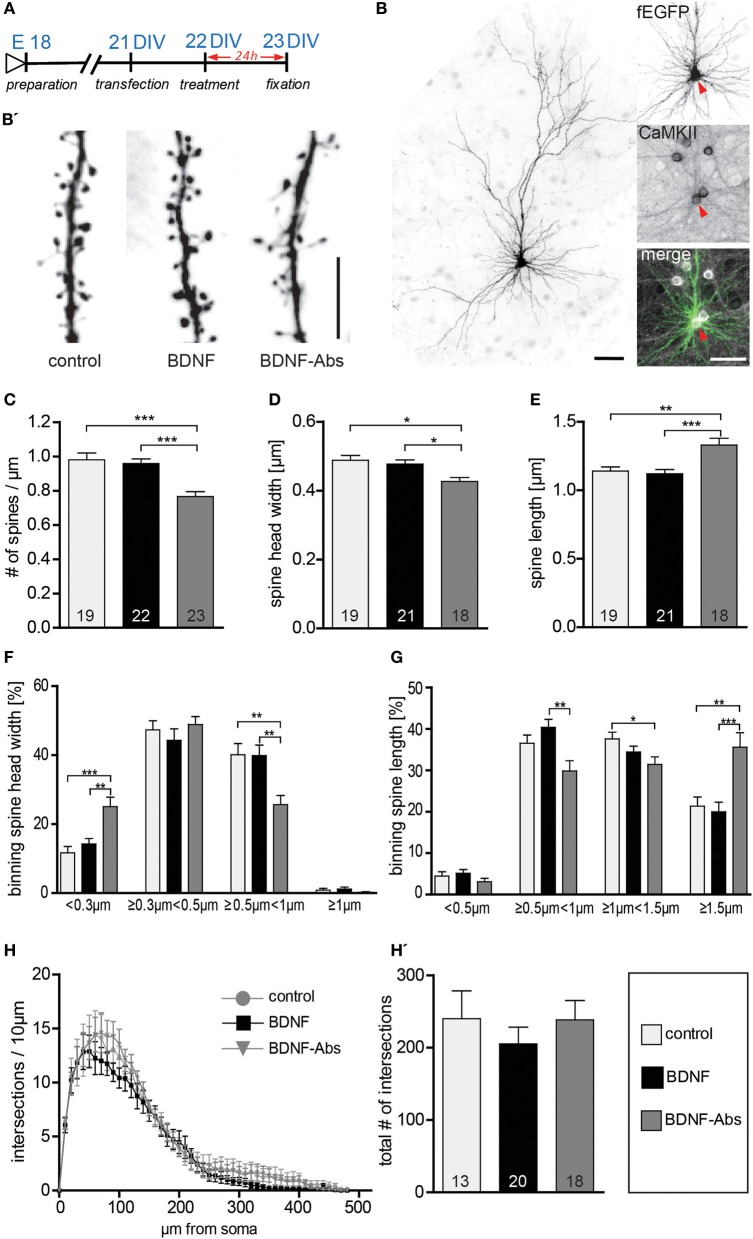
**(A)** Experimental timeline. DIV23 hippocampal neurons were treated for 24 h before fixation. **(B)** Representative image of a typical DIV23 fEGFP expressing hippocampal neuron used in the experiments; the inserts on the right show a close up of the cell body labeled with fEGFP (above) or with an immunohistochemistry against CaMKII (middle) merged to show colocalization (below): scale bars are 50 μm. **(B′)** High-magnification image of typical dendritic stretches from control (left), BDNF (middle) and BDNF antibodies (right) treated neurons. Scale bar, 5 μm. **(C)** Graphs comparing dendritic spine density between BSA (control), BDNF and BDNF antibodies (BDNF-Abs) treated DIV22 hippocampal neurons. **(D)** Histogram comparing the spine head width between hippocampal neurons upon BSA (control), BDNF or BDNF antibodies (BDNF-Abs) treatment. **(E)** Histogram of the dendritic spine length in control, BDNF or BDNF antibodies (BDNF-Abs) treated neurons. **(F)** Graph showing the binning of spines according to their spine head width and comparing the proportion of spines within each category in response to a BSA (control), BDNF or BDNF antibodies (BDNF-Abs) treatment. **(G)** Graph showing the binning of spines according to their spine length and comparing the proportion of spines within each category in response to a BSA (control), BDNF or BDNF antibodies (BDNF-Abs) treatment. **(H)** Graph plotting dendritic complexity in relation to the distance to the cell body of hippocampal neurons under control conditions or treated with BDNF or BDNF antibodies (BDNF-Abs). **(H′)** Histogram of the total number of intersections for BSA (control), BDNF and BDNF antibodies (BDNF-Abs) treated neurons. The number in the columns represents the number of cells analyzed. Significance is indicated as follows: ^*^p < 0.05; ^**^p < 0.01; ^***^p < 0.001. Error bars indicate s.e.m. ANOVA, *post-hoc* Tukey's Multiple Comparison Test.

In view of the discrepancy existing between a described action of exogenous BDNF in modulating the dendritic architecture of hippocampal neurons *in vitro* (Tyler and Pozzo-Miller, 2001; Ji et al., 2005, 2010) and previous data showing that a global deprivation of BDNF throughout the CNS *in vivo* results in only minimal morphological alterations of mature hippocampal neurons (Minichiello et al., 1999; Zakharenko et al., 2003; Rauskolb et al., 2010, for a review see Zagrebelsky and Korte, 2013), we analyzed the role of BDNF in modulating dendritic spine number and spine morphology as well as dendritic architecture of primary hippocampal neurons. The dendrite and dendritic spine architecture of mature 3-week-old (22 DIV) primary hippocampal neurons (Papa et al., 1995), expressing a membrane targeted farnesylated EGFP (fEGFP; Figure 1B) and identified as excitatory by their pyramidal like morphology (Figure 1B) and by the expression of the Ca^2+^/calmodulin-dependent protein kinase (CaMKII; Figure 1B inserts) were analyzed upon the application of either a gain- or loss-of-function approach for BDNF (Figure 1A). While 22 DIV fEGFP-expressing neurons treated for 24 h with exogenous BDNF showed no obvious alterations in dendritic spine density when compared to control neurons, (Figure 1B′), treating the neurons with BDNF function blocking antibodies (BDNF-Abs) led to a strong reduction in dendritic spine density (Figure 1B′). This observation was confirmed by the quantification of dendritic spine density showing no significant difference between BDNF and control treated cells (Figure 1C, Table S1). On the other hand, a significant reduction in dendritic spine density was observed for neurons treated with BDNF blocking antibodies (Figure 1C, Table S1; control vs. BDNF-Abs, *p* < 0.001; BDNF vs. BDNF-Abs *p* < 0.001). These observations suggest that while the endogenous BDNF may be crucial for the maintenance of dendritic spines in mature primary hippocampal neurons, exogenous BDNF does not play a role in this context. We next assessed whether BDNF regulates spine morphology in mature hippocampal neurons by analyzing the effects of adding or blocking BDNF on dendritic spine head width and length. When compared spine head width as well as spine length (Figure 1B′) did not show any major difference after a 24 h treatment with BDNF in comparison to the control condition. On the other hand neurons treated with BDNF blocking antibodies showed clearly elongated and thinner spines than control neurons (Figure 1B′). Indeed, quantification of spine morphology upon BDNF antibody treatment showed a significant decrease in spine head width (Figure 1D, Table S1; control vs. BDNF-Abs and BDNF vs. BDNF-Abs, *p* < 0.05) associated to a significant increase in dendritic spine length (Figure 1E, Table S1; control *vs*. BDNF-Abs *p* < 0.01, BDNF vs. BDNF-Abs *p* < 0.001). Treatment with exogenous BDNF did not result in any significant change in dendritic spine head width (Figure 1D, Table S1). Under these conditions dendritic spine length was only slightly reduced when compared to controls but significantly lower than in neurons treated with BDNF blocking antibodies (Figure 1E, Table S1; BDNF vs. BDNF-Abs, *p* < 0.001). Next dendritic spines were separated according on their stage of maturation into four categories based on their head width. Comparing the effect of the different treatments within each spine head category showed a significant increase in spines with a small head (<0.3 μm; control vs. BDNF-Abs *p* < 0.001, BDNF vs. BDNF-Abs *p* < 0.01) accompanied by a significant decrease in larger spine heads (≥0.5 μm <1 μm; control vs. BDNF-Abs *p* < 0.01, BDNF vs. BDNF-Abs *p* < 0.001) when a treatment with BDNF blocking antibodies was compared to a control treatment or to a treatment with exogenous BDNF (Figure 1F). Application of BDNF (gain-of-function experiment) did not result in any significant alteration in spine head size when compared to the control conditions (Figure 1F). Binning the spines according to their length did not show any difference for BDNF treated neurons but resulted in a significant alteration in spine length distribution upon BDNF antibody treatment (loss-of-function) (Figure 1G). While under these conditions spines shorter than 1.5 μm were significantly decreased (Figure 1G; ≥0.5 μm <1 μm *p* < 0.01 to the BDNF treatment; ≥1 μm <1.5 μm *p* < 0.05 to the control), those longer that 1.5 μm were significantly increased when compared to the control conditions or the BDNF treatment (Figure 1G; *p* < 0.01 to the control, *p* < 0.001 to BDNF treatment). Finally, the effects of either a gain- or a loss-of-function for BDNF on dendritic architecture were analyzed using a Sholl analysis to compare dendritic complexity. No significant differences in dendritic complexity could be observed between the three different treatments (Figure 1H; control, BDNF, BDNF-Abs). Accordingly, also total dendritic complexity was not significantly different in BDNF or BDNF- antibody-treated cells vs. control cells (Figure 1H′, Table S1).

Taken together, these results demonstrate that the exogenous application of BDNF, in mature primary hippocampal neurons, does not influence dendritic spine density, morphology and dendrite complexity. On the other hand, the loss-of-function experiments show that the endogenous BDNF is essential for maintaining spine density and the mature morphology of dendritic spines in primary hippocampal neurons.

### Endogenous BDNF regulates dendritic spine density and morphology in developing primary hippocampal neurons

**Figure 2:**
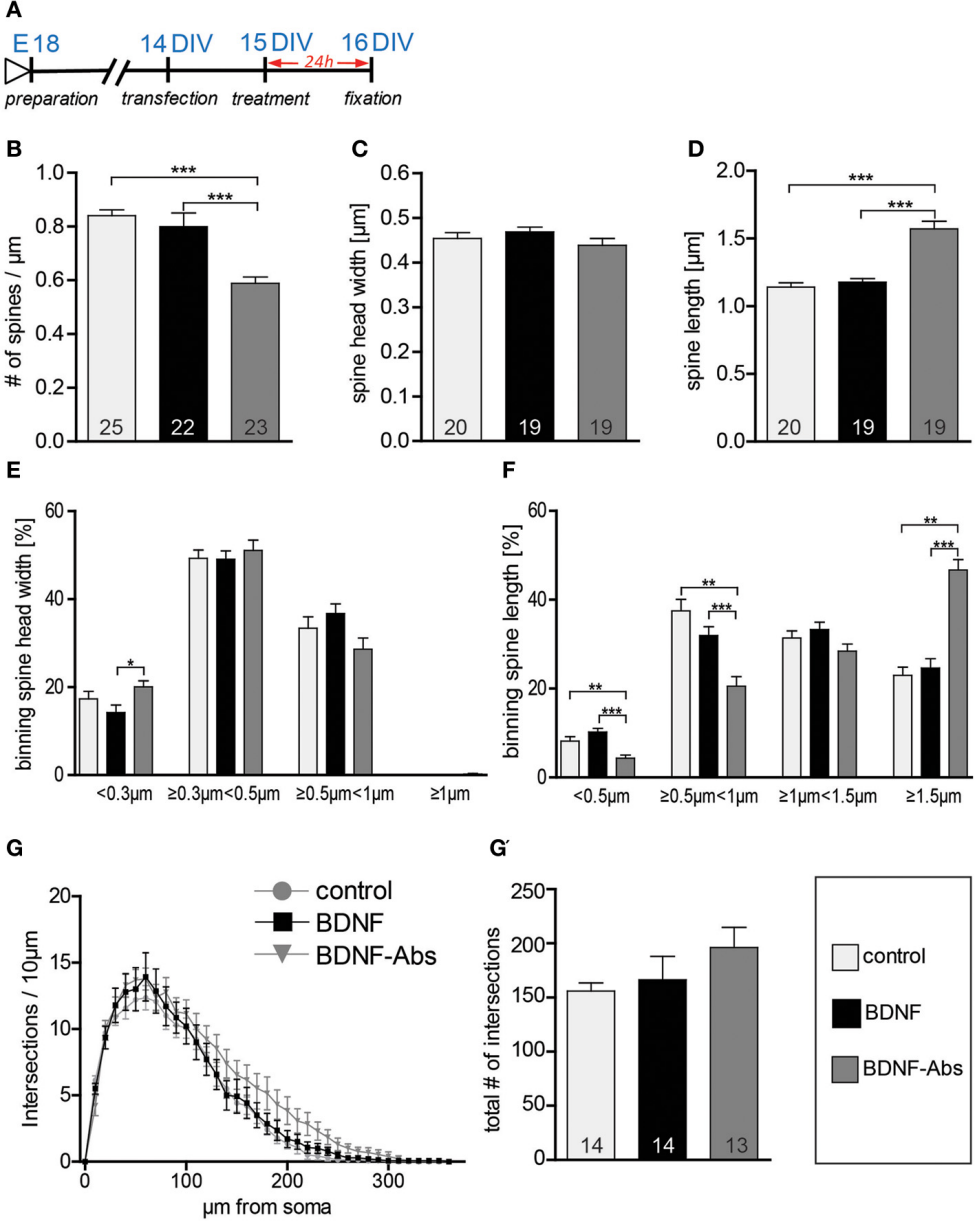
**(A)** Experimental timeline. DIV16 hippocampal neurons were treated for 24 h before fixation **(B)** Graphs comparing dendritic spine density between control, BDNF and BDNF-antibodies (BDNF-Abs) treated DIV16 hippocampal neurons. **(C)** Histogram comparing the spine head width between hippocampal neurons upon BSA (control), BDNF or BDNF antibodies treatment. **(D)** Histogram of the dendritic spine length in BSA (control), BDNF or BDNF antibodies (BDNF-Abs) treated neurons. **(E)** Graph showing the binning of spines according to their spine head width and comparing the proportion of spines within each category in response to a BSA (control), BDNF or BDNF antibodies (BDNF-Abs) treatment. **(F)** Graph showing the binning of spines according to their spine length and comparing the proportion of spines within each category in response to a BSA (control), BDNF or BDNF antibodies (BDNF-Abs) treatment. **(G)** Graph plotting dendritic complexity in relation to the distance to the cell body of hippocampal neurons under control conditions or treated with BDNF or BDNF antibodies (BDNF-Abs). **(G′)** Histogram of the total number of intersections for BSA (control), BDNF and BDNF antibodies (BDNF-Abs) treated neurons. The number in the columns represents the number of cells analyzed. Significance is indicated as follows: ^*^p < 0.05; ^**^p < 0.01; ^***^p < 0.001. Error bars indicate s.e.m. ANOVA, *post-hoc* Tukey's Multiple Comparison Test.

In the next series of experiments we tested whether the different effects of exogenous vs. endogenous BDNF on neuronal structure might depend on the age of the treated primary hippocampal neurons. To this aim we analyzed whether a gain- or a loss-of-function approach for BDNF regulates the dendritic architecture or spine number and morphology in 2 weeks-old primary hippocampal neurons (15 DIV; Figure 2A). When 15 DIV fEGFP-expressing neurons treated for 24 hours with BDNF were compared to control neurons, no obvious difference in dendritic spine density or morphology could be observed. Accordingly, the quantification of spine density did not result in any significant difference between BDNF treated and control neurons (Figure 2B, Table S1). However, as for mature hippocampal neurons, also here the application of BDNF blocking antibodies led to a highly significant reduction in dendritic spine density (Figure 2B, Table S1; control vs. BDNF-Abs and BDNF vs. BDNF-Abs *p* < 0.001). We then assessed whether BDNF regulates spine morphology comparing the maximal spine head width and spines length between different treatments. Application of exogenous BDNF for 24 h did not result in any significant alteration of the spine head width (Figure 2C, Table S1) and length (Figure 2D, Table S1) in comparison to the control condition. Interestingly, while the loss-of-function experiments did not result in any significant change in spine head width (Figure 2C, Table S1) a significant increase in dendritic spine length could be observed after application of BDNF blocking antibodies in comparison to both the control and the BDNF treatment (Figure 2D, Table S1; control vs. BDNF-Abs and BDNF vs. BDNF-Abs *p* < 0.001). Binning the spines according to their head width and comparing the different treatments within each spine head category resulted in a significant increase in the proportion of spines with a small head for the BDNF blocking antibody treatment only when compared to the BDNF treatment (Figure 2E; BDNF vs. BDNF-Abs *p* < 0.05). No significant changes could be observed for the spine head width distribution upon BDNF application (Figure 2E) when compared to the control condition. Binning the spines according to their length showed for neurons treated with BDNF blocking antibodies a significant decrease in the shorter spine category (Figure 2F; <0.5 μm, control vs. BDNF-Abs *p* < 0.01, BDNF vs. BDNF-Abs *p* < 0.001; and ≥0.5 μm <1 μm, control vs. BDNF-Abs *p* < 0.01, BDNF vs. BNF-Abs *p* < 0.001) accompanied by a significant increase in longer spines (Figure 2F; >1.5 μm, control vs. BDNF-Abs *p* < 0.01, BDNF vs. BDNF-Abs *p* < 0.001). Finally, we also analyzed whether the effect of BDNF on dendrite architecture might be age dependent. However, also for 15 DIV hippocampal neurons when either a BDNF or a BDNF antibody treatment was compared to the controls, no significant alteration in dendritic complexity could be observed for the Sholl analysis (Figure 2G). Accordingly, we did not observe any significant differences in total complexity between the different treatments (Figure 2G′, Table S1).

All in all the results described above indicate that endogenous BDNF is necessary for dendritic spine growth and to regulate spine length in developing primary hippocampal neurons. Interestingly, the spine head width in younger hippocampal neurons is not dependent on endogenous BDNF signaling.

### Endogenous BDNF regulates actin concentration within dendritic spine

**Figure 3:**
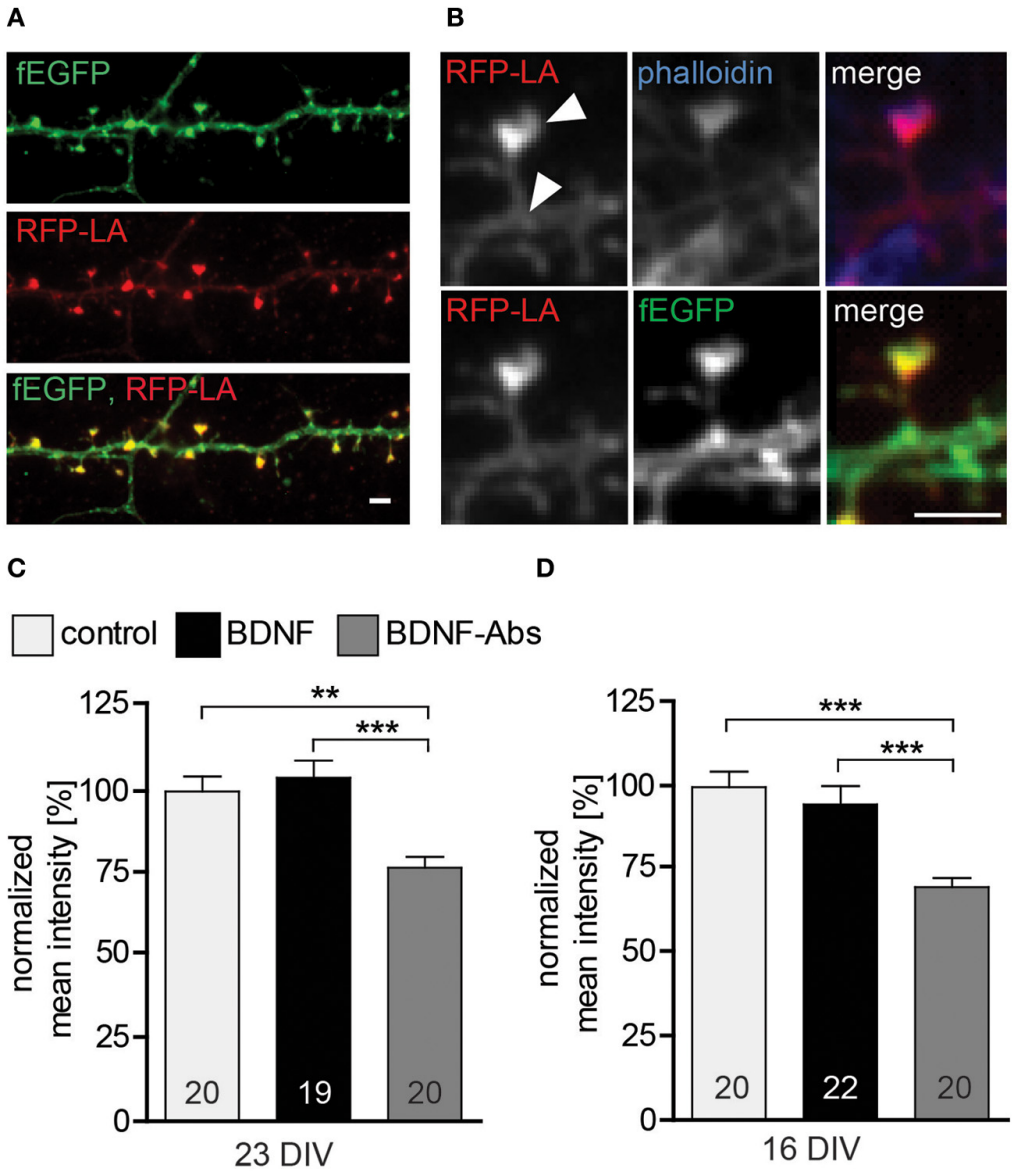
**(A)** Maximal intensity projection of a representative dendrite of hippocampal neurons expressing fEGFP (above) and RFP-Lifeact (middle) merged to show colocalization (below). Scale bar, 2 μm. **(B)** High magnification images showing a single dendritic spine expressing fEGFP (above) and RFP-Lifeact (middle) and merged to show their colocalization in the spine head (below) Scale bar, 2 μm. **(C)** Histogram plotting the normalized mean fluorescent intensity for RFP-Lifeact within the spine head of DIV23 BSA (control), BDNF and BDNF antibodies (BDNF-Abs) treated neurons. **(D)** Histogram showing the normalized mean intensity for RFP-Lifeact within the spine head of DIV16 hippocampal neurons. The number in the columns represents the number of cells analyzed. Significance is indicated as follows: ^**^p < 0.01; ^***^p < 0.001. Error bars indicate s.e.m. ANOVA, *post-hoc* Tukey's Multiple Comparison Test.

Because the actin cytoskeleton is involved in regulating cell mobility and morphology and actin is highly enriched within the dendritic spine head—for a review see (Cingolani and Goda, 2008)—we hypothesized that BDNF may regulate actin in dendritic spines in order to shape and change spine morphology. To test this hypothesis we used RFP-Lifeact (Riedl et al., 2008 and 2010) to detect possible changes in the F-actin content in dendritic spines upon changes in BDNF signaling. The RFP-Lifeact expression plasmid was co-transfected with fEGFP and resulted in 99.7% colocalization of RFP-Lifeact within spines (Figures 3A/B) as well as with F-actin labeled using Alexa Fluor 350 Phalloidin (Figure 3B). In line with our observations the exogenous application of BDNF for 24 hours to 22 or 15 DIV primary hippocampal neurons did not result in any change in the normalized mean fluorescent intensity for RFP-Lifeact in comparison to control (Figures 3C,D, Table S1). However, in the loss-of-function experiments the treatment with BDNF blocking antibodies resulted in a highly significant decrease in the F-actin content within the head of dendritic spines both in 16 DIV (Figure 3D, Table S1; control vs. BDNF-Abs *p* < 0.001, BDNF vs. BDNF-Abs *p* < 0.001) and 23 DIV primary hippocampal neurons (Figure 3D, Table S1; control vs. BDNF-Abs *p* < 0.01, BDNF vs. BDNF-Abs *p* < 0.001).

Taken together our results show that endogenous BDNF modulates the actin cytoskeleton within dendritic spines as well as their morphology.

### Mature primary hippocampal neurons are responsive to an acute treatment with exogenous BDNF

**Figure 4:**
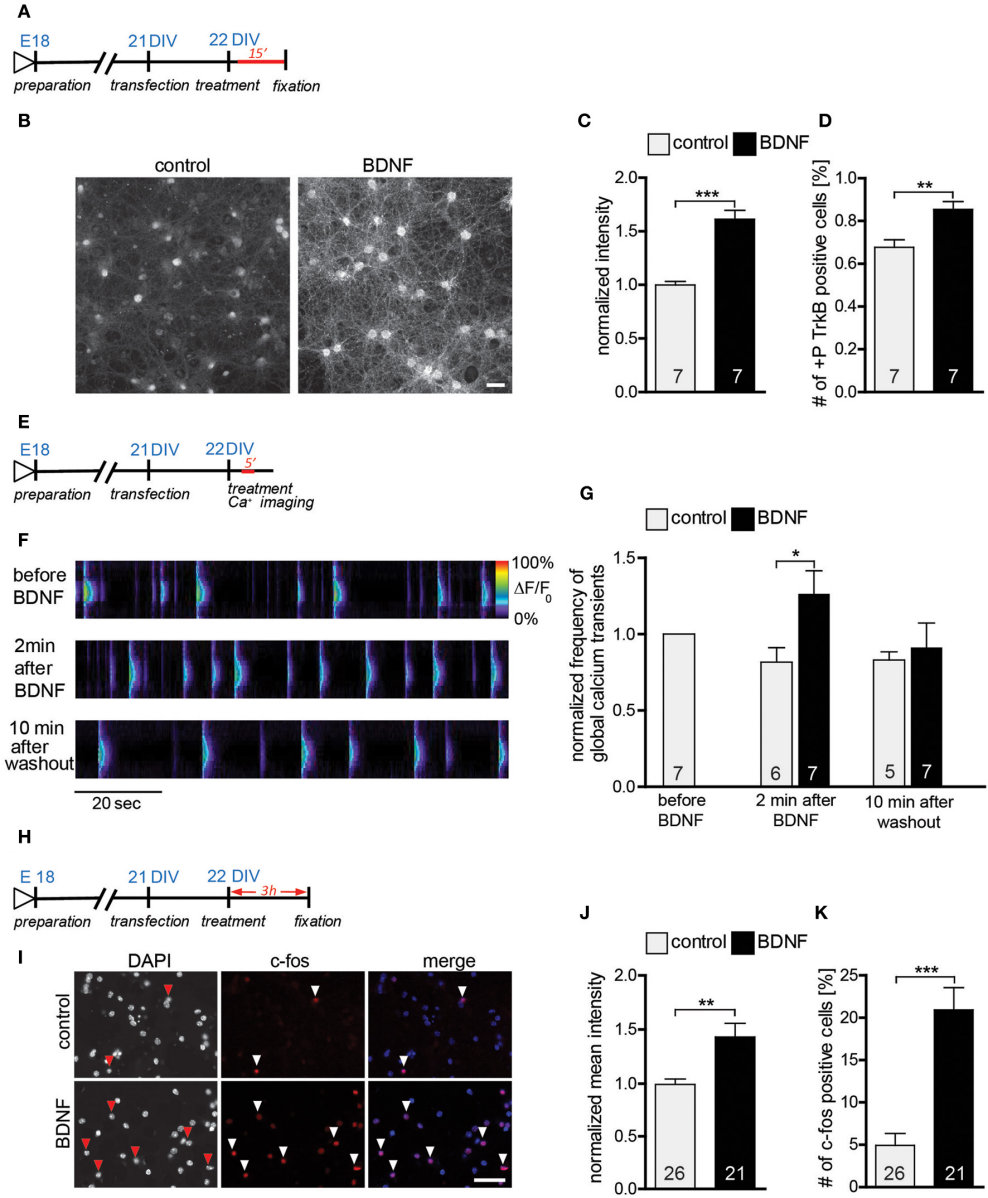
**(A)** Experimental timeline. DIV22 primary hippocampal neurons were treated 15 min with BDNF before fixation. **(B)** Images of primary hippocampal cultures (22 DIV) treated with either BSA (control) (left) or BDNF (right) and stained with an anti-phospho-TrkB receptor antibodies. Scale bar, 40 μm. **(C)** Histogram showing the normalized fluorescence intensity for the phospho-TrkB receptor under control conditions or treated with BDNF. **(D)** Graph showing the number of phospho-TrkB positive hippocampal neurons in control and BDNF treated hippocampal primary cultures. **(E)** Experimental timeline. DIV22 primary hippocampal neurons were treated 5 min with BDNF during calcium imaging. **(F)** Pseudo line-scan showing the global calcium transients occurring in hippocampal primary cultures before, during and after BDNF applications. **(G)** Graph showing the frequency of calcium transients in hippocampal primary neurons before, during and after BDNF application. **(H)** Experimental timeline. DIV22 primary hippocampal neurons were treated for 3 h with BDNF before fixation. **(I)** Images of primary hippocampal cultures (22 DIV) treated with either BSA (control) (above) or BDNF (below) and stained with an anti-c-fos antibody. Scale bar, 20 μm. **(J)** Graph comparing the normalized mean intensity for the immunohistochemistry against c-fos in control or BDNF treated hippocampal primary neurons. **(K)** Histogram of the number of c-fos positive neurons in control and BDNF treated hippocampal cultures. The number in the columns represents the number of analyzed experiments. Significance is indicated as follows: ^*^p < 0.05; ^**^p < 0.01; ^***^p < 0.001. Error bars indicate s.e.m. Unpaired two-tailed Student Test.

Our results so far clearly support a role for endogenous BDNF in the hippocampus by completely reproducing *in vitro* the mild alterations observed in the morphology of hippocampal neurons *in vivo* upon a global BDNF deprivation (Rauskolb et al., 2010). On the other hand our data fail to replicate previous work showing an effect of BDNF application in regulating dendrite and spine architecture (Tyler and Pozzo-Miller, 2001; Ji et al., 2005). To address this discrepancy we first confirmed that the BDNF used in this study is indeed active and that it is able to elicit clear neuronal responses in primary hippocampal neuronal cultures. To this aim we tested three critical events known to occur upon a BDNF application: (a) activation of the TrkB receptor was tested by analyzing its phosphorylation levels (Ji et al., 2010); (b) calcium imaging was used to test the ability of BDNF to induce local calcium transients in hippocampal neurons (Berninger et al., 1993) and (c) immunohistochemistry was used to visualize the activation of the immediate early gene (IEG) *c-fos* (Marty et al., 1996). An immunohistochemistry against phosphorylated TrkB receptor performed 15 min after BDNF application (Figure 4A) showed an obvious increase in the levels of TrkB receptor phosphorylation when compared to control treated neurons (Figure 4B). This observation was confirmed by a significant increase in the normalized intensity for the phospho-TrkB immunohistochemistry (Figure 4C, Table S2; *p* < 0.001) as well as by a clear shift in the proportion of positive vs. negative cells for phospho-TrkB. Indeed, upon BDNF treatment significantly more neurons resulted to be positive for phospho-TrkB immunohistochemistry and significantly less to be negative for it (Figure 4D, Table S2; *p* < 0.01). The activation of the PLCγ signaling pathway upon BDNF binding to the TrkB receptor leads to an increase in the intracellular calcium levels (Segal and Greenberg, 1996; Blum and Konnerth, 2005; Minichiello, 2009) and was specifically shown for hippocampal cultures (Berninger et al., 1993; Canossa et al., 1997; Finkbeiner et al., 1997). Here we analyzed the frequency of global calcium transients as readout for a neuronal response to an acute BDNF application (Figure 4E; Lang et al., 2007; Lohmann, 2009). A clear increase in the frequency of calcium transients in DIV22 primary hippocampal neurons could be observed upon the acute application of BDNF (Figure 4F). Indeed, at a quantitative analysis calcium transient frequency resulted to be significantly increased after BDNF treatment in comparison to a control treatment (Figure 4G, Table S2; BDNF vs. control *p* < 0.05). The increase in the frequency in calcium transients upon BDNF application was transient as 10 min after BDNF washout the frequency was back to the pre-treatment levels (Figure 4G, Table S2). Finally, to verify that the application of BDNF leads to the expression of immediate early genes (IEGs) we used an immunohistochemistry for c-fos known to be activated upon an acute treatment with BDNF (Figure 4H; Marty et al., 1996; Gascon et al., 2005). An immunohistochemistry for c-fos showed a clear colocalization between c-fos and the nucleus of neurons (Figure 4E). Quantitative analysis performed 3 h after the stimulation with BDNF (Figure 4H) showed significantly higher intensity values for the c-fos immunohistochemistry (Figure 4J, Table S2; *p* < 0.01) and a significant increase in the number of c-fos positive cells in comparison to control (Figure 4K, Table S2; *p* < 0.001).

In summary we could show that the BDNF used in these experiments is active and clearly leads to an activation of the TrkB-receptor, an increase in the frequency of calcium transients and an activation of transcription (up regulation of the immediate early gene *c-fos*) in mature primary hippocampal neurons.

### Exogenous BDNF regulates neurite outgrowth and complexity in young primary hippocampal neurons

**Figure 5:**
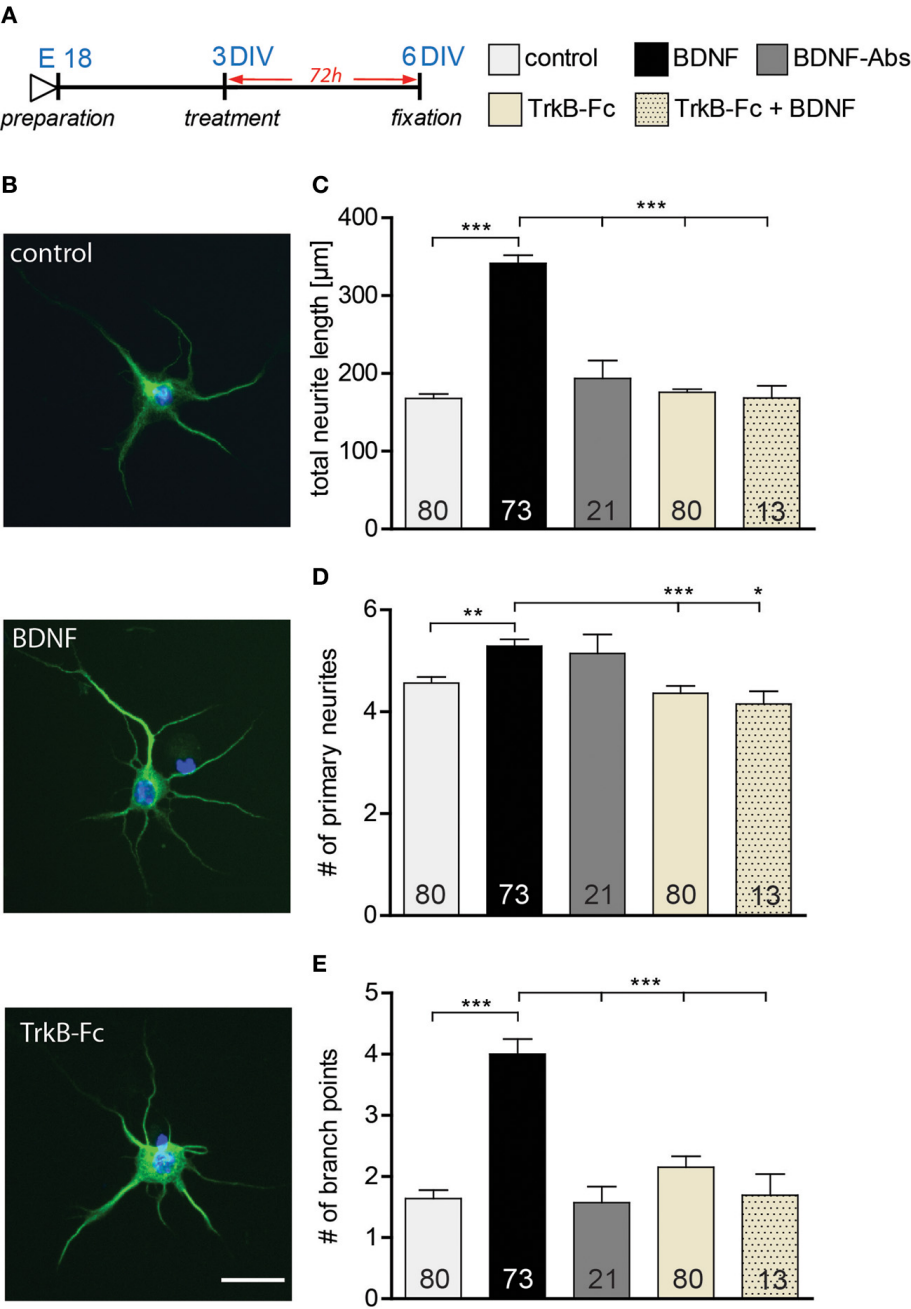
**(A)** Experimental timeline. BSA, BDNF, BDNF antibodies (BDNF-Abs) or TrkB receptor bodies (TrkB-Fc) were applied on DIV3 hippocampal neurons for 72 h. **(B)** Micrographs showing MAP2 positive BSA (control) (above), BDNF (middle) or BDNF antibodies (BDNF-Abs; below) treated primary hippocampal neurons. Scale bar, 25 μm. **(C)** Histogram comparing neurite length of DIV6 hippocampal neurons after application of BSA (control), BDNF, BDNF antibodies (BDNF-Abs), TrkB receptor bodies (TrkB-Fc) or combined BDNF and TrkB receptor bodies (TrkB-Fc). **(D)** Histogram comparing the number of primary neurites of DIV6 hippocampal neurons after application of BSA (control), BDNF, BDNF antibodies (BDNF-Abs), TrkB receptor bodies (TrkB-Fc) or combined BDNF and TrkB receptor bodies (TrkB-Fc). **(E)** Histogram comparing the number of branching points for the dendrites of DIV6 hippocampal neurons after application of BSA (control), BDNF, BDNF antibodies (BDNF-Abs), TrkB receptor bodies (TrkB-Fc) or combined BDNF and TrkB receptor bodies (TrkB-Fc). The number in the columns represents the number of cells analyzed. Significance is indicated as follows: ^*^p < 0.05; ^**^p < 0.01; ^***^p < 0.001. Error bars indicate s.e.m. ANOVA, *post-hoc* Tukey's Multiple Comparison Test.

Previous studies have shown BDNF to promote neurite outgrouth and dendritic complexity (Mcallister et al., 1995; Shimada et al., 1998). In view of the lack of effects on dendritic architecture observed in this study upon BDNF application to either DIV15 or DIV22 hippocampal primary neurons, we tested whether the effect of BDNF on dendrites might be age-dependent. Indeed, BDNF has been shown to regulate neurite outgrowth in development hippocampal neurons (Ji et al., 2005, 2010). Therefore, we treated young hippocampal neurons 3 DIV with either a gain- or a loss-of-function approach for BDNF. After 3 days of treatment (Figure 5A) an immunohistochemistry for Microtubule-associated protein 2 (MAP2) was used to visualize the developing neurites (Figure 5B; up control, middle BDNF, lower TrkB-Fc). Morphometric analysis for neurite growth was performed by quantifying three parameters: total neurite length, number of primary neurites and number of branching points (Figures 5C–E, Table S3). The total neurite length was higher after BDNF application than in control treated neurons (Figure 5C, Table S3; BDNF vs. control, *p* < 0.001). Interestingly the loss-of-function experiments with either a BDNF-Abs or TrkB-Fc did not show any significant difference in neurite length when compared to the control conditions (Figure 5C, Table S3). The specificity of the TrkB-Fc in neutralizing BDNF was determined by a co-application of BDNF and TrkB-Fc. Quantitative analysis revealed that application of TrkB-Fc completely prevented the positive effect of a BDNF treatment on the total neurite length (Figure 5C, Table S3). The number of primary neurites after BDNF application showed a slight increase when compared to the control (Figure 5D, Table S3; BDNF vs. control, *p* < 0.01), whereas the treatment with BDNF-Abs or TrkB-Fc did not alter neurite number (Figure 5D, Table S3). The significant increase in primary neurites observed after BDNF treatment could be prevented by a co-treatment with TrkB-Fc (Figure 5D, Table S3). Next we determined whether a BDNF application might affect neurite complexity by analyzing the number of branching points of MAP2 labeled processes. Indeed, a BDNF treatment resulted in a 2-fold increase in complexity in comparison to control (Figure 5E, Table S3; BDNF vs. control, *p* < 0.001). On the contrary, treatment with BDNF-Abs or TrkB-Fc did not influence the number of branching points when compared to the control (Figure 5E, Table S3). Additionally, the positive effect of BDNF on neurite complexity was prevented by a co-treatment with BDNF and TrkB-Fc (Figure 5E, Table S3).

Our results suggest that the application of BDNF promotes neurite outgrowth by increasing both neurite elongation and branching in developing DIV3 hippocampal primary neurons. Interestingly, two loss-of-function approaches for BDNF did not affect the neurite architecture of primary hippocampal neurons (3–6 DIV) during their early development.

### Role of neuronal activity in regulating the sensitivity of mature hippocampal neurons upon exogenous BDNF application

**Figure 6:**
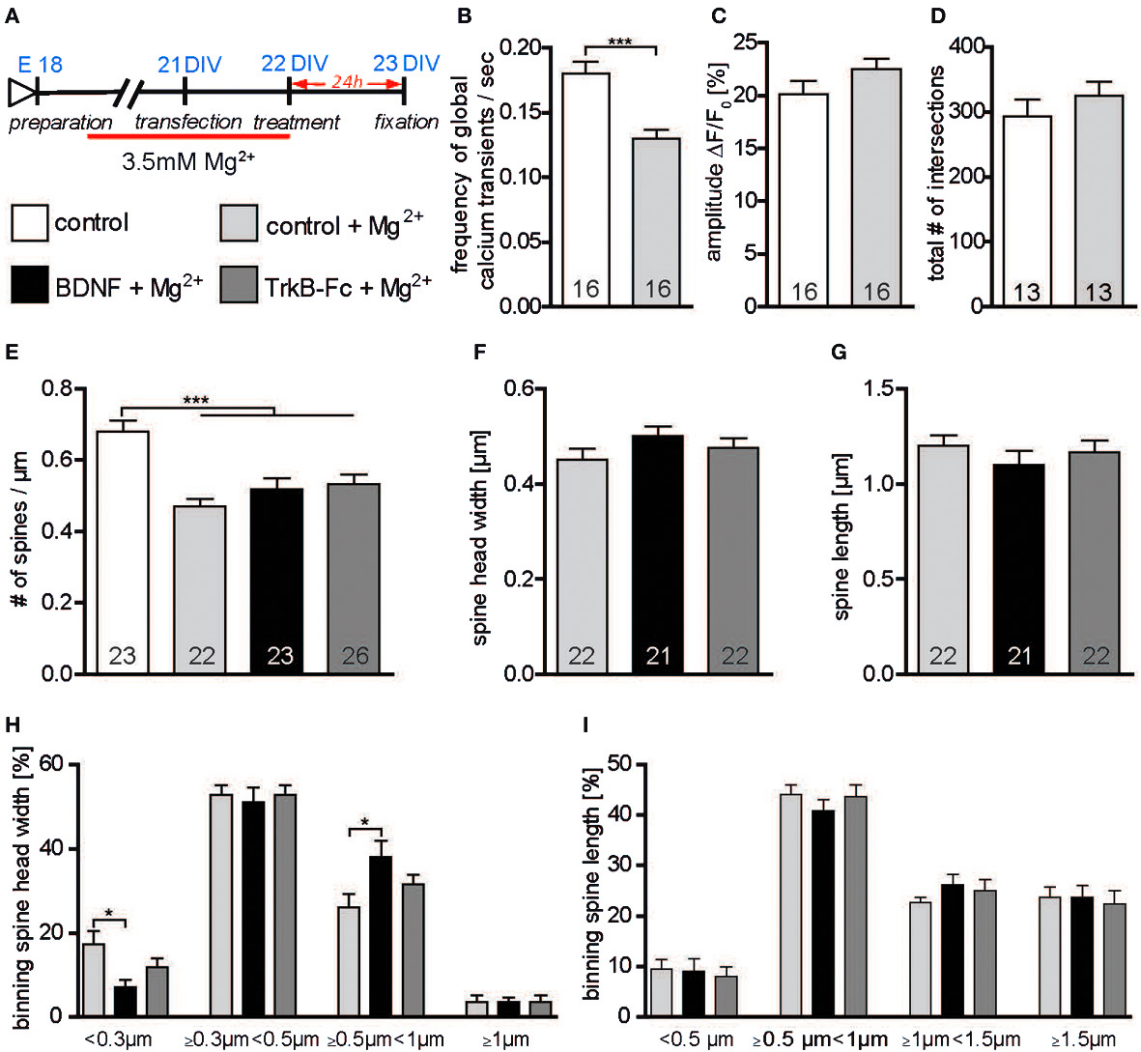
**(A)** Experimental timeline. Primary neurons were cultivated for 2 weeks in high (3.5 mM) Mg^2+^ medium and treated with BSA (control), BDNF or TrkB-Fc in low (1.5 mM) Mg^2+^. **(B)** Graph comparing the frequency of calcium transients in hippocampal primary neurons cultivated in high or low Mg^2+^ containing medium. **(C)** Graph comparing the amplitude of calcium transients in hippocampal primary neurons cultivated in high or low Mg^2+^ containing medium. **(D)** Histogram of the total number of intersections in hippocampal primary neurons cultivated in high or low Mg^2+^ containing medium. **(E)** Graphs comparing dendritic spine density between neurons cultivated in low or high Mg^2+^ and treated with BSA (control), BDNF and TrkB receptor bodies (TrkB-Fc). **(F)** Histogram comparing the spine head width between primary hippocampal neurons cultivated in high Mg^2+^ and treated with BSA (control), BDNF and TrkB receptor bodies (TrkB-Fc). **(G)** Histogram of the dendritic spine length between primary hippocampal neurons cultivated in high Mg^2+^ and treated with BSA (control), BDNF and TrkB receptor bodies (TrkB-Fc). **(H)** Graph showing the binning of spines according to their spine head width and comparing the proportion of spines within each category in response to a BSA (control), BDNF or BDNF antibodies (BDNF-Abs) treatment. **(I)** Graph showing the binning of spines according to their spine length and comparing the proportion of spines within each category in response to a control, BDNF or TrkB receptor bodies (TrkB-Fc) treatment. The number in the columns represents the number of cells analyzed. Significance is indicated as follows: ^*^p < 0.05; ^***^p < 0.001. Error bars indicate s.e.m. Unpaired two-tailed Student Test and ANOVA, *post-hoc* Tukey's Multiple Comparison Test.

BDNF synthesis and release have been shown to occur in an activity-dependent way (Thoenen, 1991; Gärtner and Staiger, 2002). Therefore, one possible explanation for the discrepancies in the effect of BDNF application on neuronal morphology *in vitro* (Ji et al., 2005, 2010; Rauskolb et al., 2010) may lie in the different levels of neuronal activity in the primary cultures. Indeed, in cultures with a higher neuronal activity the levels of endogenous BDNF may be already high and might mask the effects of BDNF application. To test this hypothesis primary hippocampal neurons were kept for 2 weeks in a medium containing a higher concentration of Magnesium (3.5 mM Mg^2+^; Figure 6A) and the effects of a 24 h treatment with BDNF (in NB medium with low, 1.5 mM Mg^2+^) on the number and structure of dendritic spines were analyzed. Calcium imaging was used to compare the levels of neuronal activity in hippocampal neurons grown under high Mg^2+^ to those of neurons grown under control (low Mg^2+^) conditions. While no significant difference could be detected regarding the amplitude of global calcium transients between the two groups (Figure 6C, Table S4), their frequency was significantly decreased in neurons kept in high Mg^2+^ when compared to control neurons (Figure 6B, Table S4; *p* < 0.001) suggesting a long-term reduction in neuronal activity. Accordingly, while dendritic complexity was unchanged (Figure 6D, Table S4), a significantly lower dendritic spine density could be observed when comparing control neurons kept in high Mg^2+^ with control neurons kept in low Mg^2+^ medium (Figure 6E, Table S4; *p* < 0.001). On the other hand, no significant differences in spine density could be detected upon a BDNF or a TrkB-Fc treatment for neurons grown under high Mg^2+^ conditions when compared to the control high Mg^2+^ conditions (Figure 6E, Table S4). Next, a possible effect of BDNF on dendritic spine morphology was analyzed. Interestingly, compared to control treated neurons BDNF treatment in cultures kept in high Mg^2+^ resulted in a slight increase in dendritic spine width (Figure 6F, Table S4) and a slight decrease in spine length (Figure 6G, Table S4), but the difference did not reach significance. No significant differences were observed both for the head width and for the length when TrkB-Fc treated cells in high Mg^2+^ were compared to high Mg^2+^ controls (Figures 6F,G, Table S4). To better clarify the mild spine head increase observed in neurons kept in high Mg^2+^ medium upon BDNF treatment spines were binned into four categories according to their head width. Comparing spine head width within each category showed a significant increase upon BDNF treatment in the proportion of spines with larger heads (Figure 6H; ≥0.5 μm <1 μm; *p* < 0.05) with a comparable significant decrease in the proportion of spine with small heads (Figure 6H; <0.3 μm; *p* < 0.05) when compared to control neurons cultivated in high Mg^2+^. No significant difference was observed between the control and TrkB-Fc treatments in any of the spine head width categories under the same conditions (Figure 6H). Comparing the effect of a control, a BDNF or a TrkB-Fc treatment within different categories of dendritic spine binned according to their length did not show any significant difference (Figure 6I). The data described above suggest that indeed a chronic reduction in neuronal activity during the cultivation time might reduce the endogenous BDNF levels in hippocampal primary neurons and thereby increase their sensitivity to an exogenous BDNF application to modulate dendritic spine morphology.

### The action of exogenous BDNF in modulating dendritic spine morphology depend on neuronal activity

**Figure 7:**
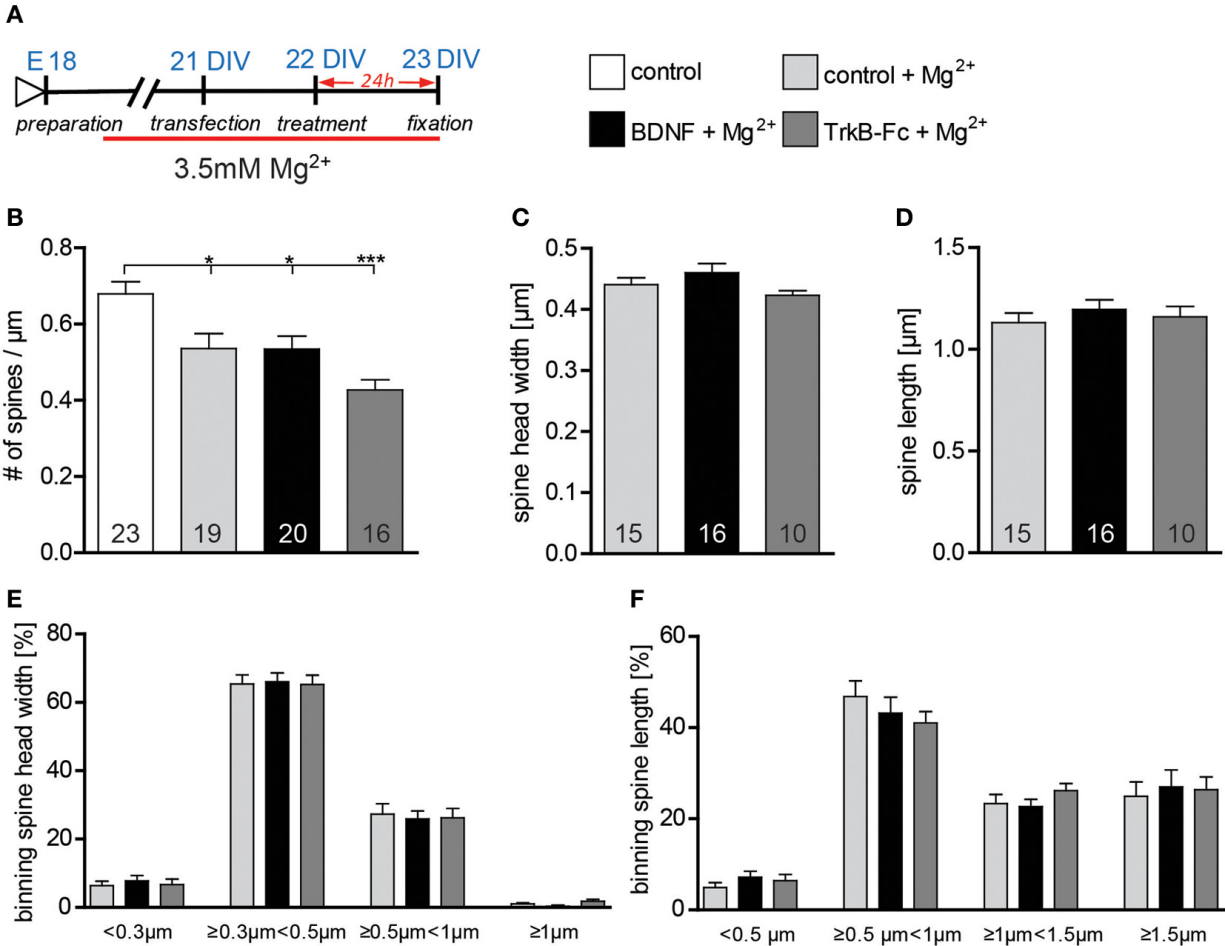
**(A)** Experimental timeline. Primary neurons were cultivated for 2 weeks in high Mg^2+^ medium and treated with BSA (control), BDNF or TrkB-Fc in high Mg^2+^. **(B)** Graphs comparing dendritic spine density between neurons cultivated in low or high Mg^2+^ and treated with BSA (control), BDNF and TrkB receptor bodies (TrkB-Fc) in high Mg^2+^. **(C)** Histogram comparing the spine head width between primary hippocampal neurons cultivated and treated in high Mg^2+^ with BSA (control), BDNF and TrkB receptor bodies (TrkB-Fc). **(D)** Histogram of the dendritic spine length between primary hippocampal neurons cultivated and treated in high Mg^2+^ with BSA (control), BDNF and TrkB receptor bodies (TrkB-Fc). **(E)** Graph showing the binning of spines according to their spine head width and comparing the proportion of spines within each category in response to a BSA (control), BDNF or BDNF antibodies (BDNF-Abs) treatment in high Mg^2+^. **(F)** Graph showing the binning of spines according to their spine length and comparing the proportion of spines within each category in response to a BSA (control), BDNF or TrkB receptor bodies (TrkB-Fc) treatment in high Mg^2+^. The number in the columns represents the number of cells analyzed. Significance is indicated as follows: ^*^p < 0.05; ^***^p < 0.001. Error bars indicate s.e.m. Unpaired two-tailed Student Test and ANOVA, *post-hoc* Tukey's Multiple Comparison Test.

BDNF has been shown to modulate dendritic spine morphology upon activity-dependent synaptic plasticity Tanaka (Tanaka et al., 2008). To assess whether under our culture condition neuronal activity governs the BDNF effect on dendritic spine morphology, hippocampal primary neurons were kept for 2 weeks and treated with BDNF in a high Mg^2+^ (3.5 mM) medium (Figure 7A) in order to reduce neuronal activity. Under these conditions a significantly lower dendritic spine density could be observed when comparing control neurons kept in high Mg^2+^ to control neurons in low Mg^2+^ medium (Figure 7B, Table S4; *p* < 0.05). Moreover, dendritic spine density of neurons treated either with BDNF or with TrkB-Fc in high Mg^2+^ was significantly lower than in controls (low Mg^2+^) neurons (Figure 7B, Table S4; BDNF vs. low Mg^2+^ control *p* < 0.05; TrkB-Fc *vs*. low Mg^2+^ control *p* < 0.001). Interestingly, both a BDNF treatment as well as a treatment with TrkB-Fc performed in high Mg^2+^ did not affect either spine head width (Figure 7C, Table S4) or spine length (Figure 7D, Table S4) when compared to a control treatment in high Mg^2+^. Also binning the spines according to their head width (Figure 7E) or to their length (Figure 7F) did not show any alteration in the spine size distributions both for a BDNF and a TrkB-Fc applications compared to a control treatment performed in high Mg^2+^.

The results above indicate that BDNF application indeed modulates dendritic spine architecture in an activity-dependent manner.

## Discussion

The main findings in this study are that the effects exerted by BDNF on the dendritic architecture of hippocampal neurons are dependent on the neuron's maturation stage and that BDNF is specifically required for the activity-dependent maintenance of the mature spine phenotype in mature excitatory hippocampal neurons *in vitro*. Indeed, our current results reproduce previous results obtained *in vivo* (Rauskolb et al., 2010). These observations further support the notion, that BDNF is a precise mediator of specific effects on neurons rather than a pleiotropic molecule unspecifically promoting cell health and survival. Indeed, besides its role in promoting neurite outgrowth (Ji et al., 2005, 2010), which we can confirm in the data presented here, BDNF is required for the activity-dependent maturation and stabilization of dendritic spines.

Our results are in line with previous work showing a surprising selectivity in the brain areas requiring BDNF-signaling for normal post-natal development (Minichiello et al., 1999; Zakharenko et al., 2003; Rauskolb et al., 2010; Zagrebelsky and Korte, 2013). Indeed, while a global BDNF deprivation throughout the CNS impairs the proper post-natal growth of striatal medium spiny neurons, BDNF deprivation fails to cause major alterations in the fine structure of mature CA1 pyramidal neurons (Rauskolb et al., 2010). These observations suggest a crucial but rather limited and specific effect of BDNF in maintaining the mature phenotype of dendritic spines without affecting the general architecture of hippocampal neurons. Our results *in vitro* show a very similar effect upon the application of two loss-of-function approaches for BDNF (function blocking BDNF antibodies, TrkB receptor bodies) confirming a physiological role of the endogenous BDNF in this context. On the other hand, our experiments fail to reproduce previously published data showing a highly significant increase in dendritic spine density and alterations in spine morphology upon the application of exogenous BDNF to mature hippocampal primary neurons (Ji et al., 2005, 2010) or to hippocampal slice cultures (Tyler and Pozzo-Miller, 2001). The discrepancy between the data presented here and other previously published data regarding the role of exogenous BDNF in regulating the architecture of mature hippocampal neurons may have different reasons. One possible explanation is the different animal species used for the experiments. While studies showing an effect of a BDNF loss-of-function either *in vivo* (Rauskolb et al., 2010) or *in vitro* (our current study) were mostly performed in mouse derived cultures, most of the previous *in vitro* work describing an effect for a BDNF gain-of-function was done in rat hippocampal neurons (Tyler and Pozzo-Miller, 2001; Ji et al., 2005, 2010) suggesting a possible difference in the sensitivity to BDNF signaling in the two different species—or simply a different level of BDNF release from neurons into the medium. Moreover, culture conditions have been shown to possibly influence the expression levels as well as the cellular response to BDNF (Chapleau et al., 2008). Indeed, here we show that the levels of neuronal activity in the cultures determine the responsiveness to the exogenous or the endogenous BDNF (Figures 6, 7). Low levels of neuronal activity during the cultivation period result in lower spine density values and an increased responsiveness of the hippocampal neurons to the applications of exogenous BDNF. Indeed, the synthesis and secretion of BDNF have been shown to be directly controlled by neuronal activity (reviewed in Thoenen, 1991; see also Gärtner and Staiger, 2002). Accordingly, control neurons in previous publications showing a strong effect upon a BDNF application (Ji et al., 2005, 2010) had a spine density which is about 10 times smaller than the one measured in the current study or described *in vivo* (De Simoni et al., 2003) and 5 times smaller than the one described for a more complex *in vitro* system (De Simoni et al., 2003; Rauskolb et al., 2010). It should also be noted that in our culture system the levels of neuronal activity as well as the cellular responsiveness to BDNF were tested in different ways (Figures 4A–K). Application of exogenous BDNF has been shown to trigger a direct response of the neuronal network via a transient elevation of the intracellular calcium concentration (Berninger et al., 1993; Lang et al., 2007). In contrast, blocking intrinsic BDNF signaling was shown to reduce the frequency of spontaneously occurring calcium rises in developing rat hippocampal neurons (Lang et al., 2007). In our study calcium imaging was used first to test the responsiveness of the hippocampal neurons to BDNF and also to test the effects on neuronal activity of manipulating the Mg^2+^ concentration in the culture medium. Thereby we could confirm that increasing Mg^2+^ concentration in the medium results in a long lasting reduction in neuronal excitability and network activity of primary hippocampal cultures (Dribben et al., 2010). While this reduction in neuronal activity does not influence dendritic morphology and complexity, it's accompanied by a significant decrease in dendritic spine density as well as by an increased cellular sensitivity to the application of exogenous BDNF. The cellular responsiveness to BDNF was tested also quantifying the changes in TrkB phosphorylation as well as the activation of the IEG c-fos, two critical events known to occur upon the acute activation of BDNF signaling (Marty et al., 1996; Ji et al., 2005, 2010). Our results indicate that in spite of the lack of morphological effects of an application of BDNF, mature excitatory, hippocampal neurons under the culture conditions used in this work are responsive to BDNF. In addition, their responsiveness is increased under low neuronal activity conditions. Whether the increase neuronal responsiveness to BDNF is due to an activity-dependent decrease in BDNF release from neurons or, as recently described from microglia (Parkhurst et al., 2013) will be very interesting to address. Indeed, changes in magnesium concentration have been shown to possibly influence ATP release (Li et al., 1997) and could thereby indirectly influence BDNF release from microglia (Trang et al., 2009; Parkhurst et al., 2013). Moreover, it will be crucial to explore the possibility that different amounts of microglia may influence the BDNF levels in the primary hippocampal cultures and thereby their responsiveness to a BDNF exogenous application.

Our results show a requirement for neuronal-activity during the application for BDNF to exert its effect on dendritic spines. This observation is in line with previous work showing that during the early development of cortical pyramidal neurons in ferrets BDNF regulates dendritic architecture in an activity-dependent manner (Mcallister et al., 1996). Moreover, in cultures of cerebellar neurons, BDNF increases the spine density of Purkinje cells only in the presence of granule cells suggesting a crucial role of neuronal activity in mediating this BDNF effect (Shimada et al., 1998). In line with this observation is the role of BDNF in regulating structural changes at dendritic spines upon activity-dependent synaptic plasticity in mature hippocampal neurons (Tanaka et al., 2008).

One important open question concerns the functional consequences of the observed structural changes at spines regulated by BDNF signaling. Our results upon a BDNF loss-of-function confirm the structural alterations at spine observed already *in vivo* (Rauskolb et al., 2010). Specifically, a BDNF deprivation results in a decrease in dendritic spine density accompanied by a decrease in spine head width and an increase in spine length suggesting a loss of the mature phenotype of spines possibly resulting in a decrease and a weakening of synaptic connections under these conditions. Indeed, spine head width is correlated with the size of the post-synaptic density and the number of AMPA receptors (Holtmaat and Svoboda, 2009) and a shrinking of the spine head width has been correlated to the synaptic weakening occurring upon long term depression induction (Zhou et al., 2004). Moreover, the spine neck has been suggested to act as a diffusion barrier regulating the biochemical compartmentalization in the spine head (Sjostrom et al., 2008; Holthoff et al., 2010). Indeed, spines with long necks have been shown to be electrically silent at the soma, although their heads are activated by an uncaging event (Araya et al., 2006) and a BDNF-dependent shortening of the spine neck was observed upon inducing long term potentiation at single spines (Tanaka et al., 2008). It has been show that upon maturation the proportion of mushroom spines increases while the one of thin long spines decreases (De Simoni et al., 2003). Taken together these data suggest that BDNF signaling plays a crucial role in maintaining the mature architecture of dendritic spines.

Finally, we show in our experiments that endogenous BDNF modulates the actin cytoskeleton within dendritic spines as well as their morphology. This is of interest with respect to the fact that activity-dependent as well as spontaneous structural changes of dendritic spines are dependent on the modulation of actin dynamics within spines (Krucker et al., 2000; Fukazawa et al., 2003; Kramar et al., 2004). Noteworthy in this context is the observation that BDNF modulates actin regulatory binding proteins which control the turnover of the actin cytoskeleton as well as **c**ytoskeleton-associated proteins like Arc/Arg3.1 (Ying et al., 2002; Messaoudi et al., 2007; Rex et al., 2007; Bramham, 2008). Interestingly, while a gain-of-function approach performed in this study did not affect the F-actin content within dendritic spines, a loss-of-function experiment resulted in a significant decrease in polymerized actin (see Figure 3). This is in line with the results of Rex et al. (2007) which show that exogenous application of BDNF to rat hippocampal slices increased the number of F-actin labeled spines only when theta burst stimulation was applied (Rex et al., 2007). Moreover, the effect of theta burst stimulation on F-actin was completely abolished by a BDNF scavenger (TrkB-Fc; Rex et al., 2007) suggesting a role for the TrkB receptor in this context. On the other hand, BDNF might also modulate actin dynamics via the p75 Neurotrophin Receptor (p75^NTR^). In a series of gain- and loss-of-function experiments we previously showed that the p75^NTR^ negatively modulates dendritic spine density and morphology (Zagrebelsky et al., 2005), in addition to its effect on long-term depression (Rösch et al., 2005; Woo et al., 2005). It has been shown that signaling via the p75^NTR^ controls the activity of the small GTPase RhoA, thereby regulating the actin cytoskeleton (Yamashita et al., 1999; Yamashita and Tohyama, 2003; Gehler et al., 2004) and possibly preparing it for processes of negative synaptic plasticity.

In summary our study reports highly specific effects of BDNF on the maintenance of mature spines in excitatory hippocampal neurons and it also shed some light on why published *in vivo* and *in vitro* results concerning morphological effects of BDNF are often contradictory.

### Conflict of interest statement

The authors declare that the research was conducted in the absence of any commercial or financial relationships that could be construed as a potential conflict of interest.
