# Supplementary material for: The BDNF effects on dendritic spines of mature hippocampal neurons depend on neuronal activity
Source: Front Synaptic Neurosci. 2014 Mar 20;6:5. doi: 10.3389/fnsyn.2014.00005 (PMC3960490; doi:10.3389/fnsyn.2014.00005)
Supplement: Supplementary file 1 [file DataSheet1.DOCX]

**Tables**

**Table S1**

The table reports the values for spine density, head width and length as well as the total number of dendritic intersections for control, BDNF and BDNF-Abs treated neurons for the experiments described in **Fig.1** and **2** (above and middle). The values for the normalized mean intensity for Lifeact of control, BDNF and BDNF-Abs treated neurons for the experiments described in **Fig.3** are given in the lower part.

| Figure 1. | Control | BDNF | BDNF-Abs |
| --- | --- | --- | --- |
| C. # of spines / µm | 0.98 ± 0.04 (n=19) | 0.96 ± 0.03 (n=22) | 0.77 ± 0.03 (n=23) |
| D. spine head width [µm] | 0.49 ± 0.02 (n=19) | 0.48 ± 0.01 (n=21) | 0.43± 0.01 (n=18) |
| E. spine length [µm] | 1.14 ± 0.03 (n=19) | 1.12 ± 0.03 (n=21) | 1.33 ± 0.05 (n=18) |
| H’. total # of intersections | 240.08 ± 38.51 (n=13) | 205.25 ± 23.20 (n=20) | 238.50 ± 26.81 (n=18) |
| Figure 2. | **Control** | **BDNF** | **BDNF-Abs** |
| B. # of spines /µm | 0.84 ± 0.02 (n=25) | 0.80 ± 0.05 (n=22) | 0.59 ± 0.02 (n=23) |
| C. spine head width [µm] | 0.45 ± 0.01 (n=20) | 0.47 ± 0.01 (n=19) | 0.44 ± 0.02 (n=19) |
| D. spine length [µm] | 1.14 ± 0.03 (n=20) | 1.18 ± 0.03 (n=19) | 1.57 ± 0.06 (n=19) |
| G’. total # of intersections | 155.93 ± 7.51 (n=14) | 196.15 ± 18.44 (n=14) | 166.29 ± 21.82 (n=13) |
| Figure 3. | **Control** | **BDNF** | **BDNF-Abs** |
| C. 23DIV normalized mean intensity [%] | 100.00% ± 4.46 (n=20) | 104.16% ± 5.08 (n=19) | 76.93% ± 3.16 (n=20) |
| D.16DIV normalized mean intensity [%] | 100.00% ± 4.63 (n=20) | 94.91% ± 5.34 (n=22) | 69.97% ± 2.74 (n=20) |

**Table S2**

The table reports the values relative to the experiments described in **Fig.4**. Specifically, the normalized intensity for the phospho-TrkB immunohistochemistry and the number in % of phospho-TrkB positive cells for control and BDNF treated neurons; the values for the normalized frequency of global calcium transients for control and BDNF treatments as well as the normalized mean intensity and the number of c-fos positive cells for control and BDNF treated neurons. The numbers in brackets indicate respectively the number of experiments.

| Figure 4. |  | Control | BDNF |
| --- | --- | --- | --- |
| C. normalized intensity |  | 1.00 ± 0.03 (n=7) | 1.61 ± 0.08 (n=7) |
| D. # of +P TrkB positive cells [%] | | 0.68 ± 0.04 (n=7) | 0.85 ± 0.04 (n=7) |
| G. normalized frequency of global calcium transients | **2 min after BDNF** | 0.82 ± 0.1(n=6) | 1.26 ± 0.15 (n=7) |
|  | **10 min after washout** | 0.83 ± 0.05 (n=5) | 0.91 ± 0.16 (n=7) |
| J. normalized mean intensity | | 1.00 ± 0.06 (n=26) | 1.46 ± 0.13 (n=21) |
| K. # of c-fos positive cells [%] | | 4.92 ± 1.43 (n=26) | 20.93 ± 2.63 (n=21) |

**Table S3**

The values for total neurite length, the number of primary neurites and the number of branching points at neurites are shown for control, BDNF, BDNF-Abs, TrkB-Fc and TrkB-Fc + BDNF treated neurons for the experiments described in **Fig.5**. The numbers in brackets indicate the number of cells analyzed.

| Figure 5. | Control | BDNF | BDNF-Abs | TrkB-Fc | TrkB-Fc + BDNF |
| --- | --- | --- | --- | --- | --- |
| C. total neurite length [µm] | 167.73 ± 5.72 (n=80) | 341.43 ± 10.34 (n=73) | 193.56 ± 22.81 (n=21) | 175.71 ± 4.24 (n=80) | 168.51 ± 15.50 (n=13) |
| D. # of primary neurites | 4.56 ± 0.12 (n=80) | 5.29 ± 0.14 (n=73) | 5.14 ± 0.37 (n=21) | 4.36 ± 0.15 (n=80) | 4.15 ± 0.25 (n=13) |
| E. # of branch points | 1.64 ± 0.14 (n=80) | 4.00 ± 0.25 (n=73) | 1.57 ± 0.26 (n=21) | 2.15 ± 0.18 (n=80) | 1.69 ± 0.35 (n=13) |

**Table S4**

The table reports the values of the frequency of global calcium transients per second and the total number of intersections for control 1.5mM Mg^2+^ and control 3.5mM Mg^2+^ neurons shown in **Fig.6.** Spine density, the mean values for the spine head width and for spine length are shown for control neurons in 1.5mM Mg^2+^, control, BDNF and TrkB-Fc treated neurons in 3.5mM Mg^2+^ for the experiments described in **Fig.6** and **Fig.7.**

| Figure 6. | Control 1.5 mM Mg^2+^ | control 3.5 mM Mg^2+^ | BDNF 3.5 mM Mg^2+^ | TrkB-Fc 3.5 mM Mg^2+^ |
| --- | --- | --- | --- | --- |
| B. frequency of global calcium transients / sec | 0.18 ± 0.01 (n=16) | 0.13 ± 0.01 (n=16) |  |  |
| C. amplitude ∆F/F_0_ [%] | 20.16 ± 1.26 (n=16) | 22.51 ± 0.99 (n=16) |  |  |
| D. total # of intersections | 292.92 ± 26.28 (n=13) | 324.46 ± 22.05 (n=13) |  |  |
| E. # of spines / µm | 0.68 ± 0.03 (n=23) | 0.47 ± 0.02 (n=22) | 0.52 ± 0.03 (n=23) | 0.53 ± 0.03 (n=26) |
| F. spine head width [µm] |  | 0.45 ± 0.02 (n=22) | 0.50 ± 0.02 (n=21) | 0.48 ± 0.02 (n=22) |
| G. spine length [µm] |  | 1.20 ± 0.05 (n=22) | 1.1 ± 0.07 (n=21) | 1.17 ± 0.06 (n=22) |
| Figure 7. | **Control 1.5 mM Mg^2+^** | **Control 3.5 mM Mg^2+^** | **BDNF 3.5 mM Mg^2+^** | **TrkB-Fc 3.5 mM Mg^2+^** |
| B. # of spines / µm | 0.68 ± 0.03 (n=23) | 0.54 ± 0.04 (n=19) | 0.53 ± 0.03 (n=20) | 0.43 ± 0.03 (n=16) |
| C. spine head width [µm] |  | 0.44 ± 0.01 (n=15) | 0.46 ± 0.02 (n=16) | 0.42 ± 0.01 (n=10) |
| D. spine length [µm] |  | 1.13 ± 0.05 (n=15) | 1.20 ± 0.05 (n=16) | 1.16 ± 0.05 (n=10) |
